# Integrating ethics in public health education: the process of developing case studies

**DOI:** 10.1186/s40985-015-0002-3

**Published:** 2015-05-29

**Authors:** Theodore Tulchinsky, Bruce Jennings, Sarah Viehbeck

**Affiliations:** 1 Braun School Public Health, Hebrew University-Hadassah, Ein Karem, Jerusalem, Israel; 2 Center for Humans and Nature, Dobbs Ferry, New York; 3 Yale School of Public Health, New Haven, Connecticut USA; 4 Evaluation and Strategic Initiatives, Canadian Institutes of Health Research-Institute of Population and Public Health, Waterloo, Ontario Canada; 5 University of Waterloo, Waterloo, Ontario Canada; 6 School of Health Sciences, Ashkelon College, Ashkelon, Israel

**Keywords:** Public health ethics, Public health education, Case studies

## Abstract

The study of ethics in public health became a societal imperative following the horrors of pre World War II eugenics, the Holocaust, and the Tuskegee Experiment (and more recent similar travesties). International responses led to: the Nuremberg Doctors’ Trials, the Universal Declaration of Human Rights (1948), and the Convention on Prevention and Punishment of the Crime of Genocide (CCPCG, 1948), which includes sanctions against incitement to genocide. The Declaration of Geneva (1948) set forth the physician’s dedication to the humanitarian goals of medicine, a declaration especially important in view of the medical crimes which had just been committed in Nazi Germany. This led to a modern revision of the Hippocratic Oath in the form of the Declaration of Helsinki (1964) for medical research ethical standards, which has been renewed periodically and adopted worldwide to ensure ethical research practices. Public health ethics differs from traditional biomedical ethics in many respects, specifically in its emphasis on societal considerations of prevention, equity, and population-level issues. Health care systems are increasingly faced with the need to integrate clinical medicine with public health and health policy. As health systems and public health evolve, the ethical issues in health care also bridge the gap between the separation of bioethics and public health ethics in the past. These complexities calls for the inclusion of ethics in public health education curricula and competencies across the many professions in public health, in the policy arena, as well as educational engagement with the public and the lay communities and other stakeholders.

## Introduction

The 20th century provided horrendous examples of violence, neglect, injustice, suffering and death that resulted from disregard of fundamental values and goals of public health (PH). This was characterized by genocide based on eugenics and Nazi extreme political racist ideologies with the industrialized murder of millions of innocents in “ethnic purity” programs. These travesties demonstrated the importance of clarifying, strengthening, and promulgating the humanity, justice, and equality inherent in the ethical practices of public health. Following the Holocaust of World War II, the world responded in various ways by setting new standards of international law, human rights, and health ethics, which initially grew out of the Nuremberg Trials on war crimes and abusive medical research.

The outcomes of this international ethical reassessment have been codified in various United Nations charters of human rights and genocide, as well as in setting standards on human subject research like those set forth by the World Medical Association in the Declaration of Helsinki [1]. In the United States lessons from the Tuskegee syphilis experiments led to the Belmont Report, which outlined ethical principles of biomedical and behavioral research [2, 3]. In the ongoing legacy of these ethical standards, the education of public health professionals in public health ethics has a distinct and important role. Moreover, continuing failings and problems, such as the failure in some areas and policies to adopt evidence-based public health measures, reinforce the importance of high ethical standards in the public health profession [4]. However, until recently, the field has been slow to meet this educational responsibility.

## Review

Public health ethics is only one facet of societal ethics education more generally. Ethics education should not be restricted to the public health workforce but should be part of education in many fields of higher education, as well as in primary and secondary education. The rationale for ethics education in public health depends on the proposition that disagreements and conflict are inevitable in public health policy and practice. However, disagreements and conflicts can be reduced and overcome. Common rules and values for cooperative and peaceful living are achievable despite being faced with new religious and racist based ideologies well into the 21^st^ century.

Ethics should be an integral consideration in developing training objectives and competencies for public health professionals, taking into account societal values; scientific evidence; and the socio-cultural, political, and economic contexts. Development of educational material for public health ethics requires addressing not only formal education in public health bachelor’s and master’s degree programs, but also in medical, nursing, and social science-related fields including economics and social work. In addition, educational materials should be developed with a focus on the the broader community in general.

As with any education program, regardless of the subject matter, achieving effective training and competencies when discussing PH ethics requires careful planning and some background preparation by the discussion leader or facilitator. Educators who have a good grasp of the issues on all sides of a question (whatever their own personal convictions might be) are in a position to guide the discussion so that the participants are led not only to discover and express ethical issues themselves but also to think thoroughly about them. Moreover, it is important to familiarize teachers of public health with strategies for analyzing ethical questions in concrete cases or situations. One such strategy that has been developed and widely used in bioethics and PH ethics education during the past few years places a focus on the analysis of particular fact patterns, or “cases”, which lend themselves to an analysis of the ethical dimension of the actions and decisions that were made or should have been made in that case.

In this article we address the development and integration of ethics in formal public health degree programs and in continuing professional education in public health. Offering ethics training, including ethical analysis of the public health issues involved, should be part of every public health curriculum as a dedicated course of cross disciplinary importance. Ethics should also be incorporated in teaching material of other courses with a pervasive strategy in which core components of ethical reasoning are built into virtually every course in the entire curriculum. As identified in the other articles in this issue of *Public Health Reviews*, case studies are one of the key elements for PH ethics training and education. Case studies serve to illustrate ethical conflicts that frequently challenge policy makers and professional public health personnel. We address the development process of case studies, their peer review, and their utility in preparing faculty for incorporation of PH ethics educational components at all levels of public health education, including education of the general public [5].

### Ethics curriculum development with use of cases

In the United States, the Association of Schools of Public Health (ASPH), in collaboration with The Hastings Center and the Health Resources and Services Administration (HRSA), published a Model Curriculum on Public Health Ethics in 2004 [6]. The ASPH review approached ethics education based on analysis and discussion of real world public health case studies which raise issues relevant in the United States and globally.

The ASPH model curriculum has 10 modules. Each module has a consistent format, which consists of a background issue essay, a fact sheet, three cases for discussion based on real incidents, an analytic guide for instructors leading the group discussion of the case, and a bibliography for further reading. The curriculum’s overall introduction addresses general techniques and issues concerning the challenges of teaching the subject matter of ethics and values.

The ASPH curriculum as a whole and each module within it were designed to be more like an instructor’s manual than a textbook. Each module was written primarily for faculty members or volunteer facilitators of in-service programs. These individuals were not necessarily experts or individuals with special training in ethics, but they had some prior interest and motivation in the subject. How much of the material to share with students or participants in in-service continuing education sessions is left to the discretion of the group instructor.

The European Association of Schools of Public Health (ASPHER) in 2007 published a Working Paper on the Mission, Values and Ethics of Public Health, which provided a context and stimulated interest in discussion of PH ethics [7]. This raised the topic of public health ethics and ASPHER initiated the Working Group on PH ethics in conjunction with the European Public Health Association (EPHA) in 2009 leading to a 2012 issue and this follow-up issue of *Public Health Reviews*.

The World Health Organization (WHO) in 1999 published case studies in public health research which addressed the associated ethical issues [8]. A few years later the Public Health Ethics Unit of the US Centers for Disease Control and Prevention (CDC) included an approach to public health ethics education and training focused primarily on in-service public health workforce training programs. It is entitled, “Good Decision Making in Real Time: Public Health Ethics Training for Local Health Departments” and consists of separate materials designed for group discussion facilitators and for public health students in the training program [9].

In 2011, the Public Health Agency of Canada funded the National Collaborating Centre for Healthy Public Policy in the *Institut national de de santé publique du Québec* to “bring the emerging body of knowledge to public health actors across Canada thereby increasing their expertise to make programs and policy more responsive, just, and effective” [10] and a policy paper dealing with ethical issues related to pandemics [11]. In 2012, *Population and Public Health Ethics: Cases from Research, Policy, and Practice* published by the University of Toronto Joint Centre for Bioethics [12] compiled 16 realistic cases from across public health research, policy, and practice contexts in Canada and internationally. Case studies covered a range of topics, including outdoor smoke-free policies, mandatory immunization policies for health workers, malaria control initiatives, and inequities in health for aboriginal populations. All cases were peer-reviewed through a standard process and followed a similar format: background and context, a description of the case, a “scenario shift” that was designed to consider how the initial review of the case would change under different or changing circumstances, and discussion of related questions. A largely Canadian effort, this book extended the work of earlier casebooks by including example analyses for each of the cases written by individuals with expertise in both ethics and public health, which provides practical issues as food for thought, discussion, and debate. The casebook is being used as a resource in several public health courses in Canada and the United States.

Building on the Canadian effort, the US CDC formed an international partnership to develop new case study materials useful for public health ethics education. This is called The Public Health Ethics International Collaboration Steering Group (PHEICSG) and is composed of representatives from the following agencies: CDC; the Ethics Section of the American Public Health Association (APHA); the Canadian Institutes of Health Research (CIHR); the Pan-American Health Organization (PAHO); the World Health Organization (WHO); the European Commission (EC); the European Public Health Association (EPHA); and the Training Programs in Epidemiology and Public Health Interventions Network (TEPHINET).

This group concluded that although the practice of public health has always involved consideration of ethical issues, the field of public health ethics as a discipline is a relatively new and emerging area. Despite previous efforts relatively few practical training resources for public health practitioners are available, especially resources that consider ethical issues likely to arise in the practice of public health. The casebook assembles a broad range of cases that highlight global perspectives on the ethical challenges of public health and approaches specifically designed for addressing these challenges.

The purpose of the PHEICSG casebook is to raise awareness and provide teaching material for the understanding of public health ethics and the value of ethical analysis in public health practice. This includes, but is not limited to, ethical considerations for public health policy development, implementation, and evaluation, public health decision making in national and international field settings and training programs, and applied public health research. The cases highlight ethical issues and dilemmas that arise in the practice of public health, drawing attention to similarities and differences in cross-cultural perspectives on frequently encountered public health ethics concerns. Finally, the casebook is a tool to support instruction, debate, and dialogue regarding public health ethics that delineates approaches specifically designed to address ethical challenges encountered in public health practice.

The primary audiences for the casebook is public health practitioners, including front-line workers, field epidemiology trainers and trainees, managers, planners, and decision makers who have a responsibility and interest in learning about how to integrate ethical analysis into their day to day public health practice. The casebook is also useful to schools of public health and public health students, as well as to academic ethicists who can use the book to teach public health ethics and distinguish it from clinical and research ethics.

The format of the cases consists of a background section that provides context regarding the public health selected topic, a description of the case, and up to five questions to stimulate discussion of the ethical aspects of the case. The CDC casebook will also include introductory information on public health ethics and several chapters providing overview ethical analyses highlighting issues raised by the cases. The intended use includes public health courses or workshops on public health ethics in various settings. The casebook is expected to be published in 2015.

Ethics analysis typically involves six steps or objectives, including the capability to:

- identify the ethical problem(s) germane to the case;
- assess the factual information available to the agents and decision makers;
- identify the “stakeholders” in the case—those whose rights and interests will be most directly affected by the decisions made;
- identify the values at stake in the case;
- identify the options available to key agents or decision makers in the case; and
- assess the process for making the decision and the values that pertain to the process; before, during and after a public health event or process as in pandemic preparedness [5, 13].

This type of ethics analysis can be used retrospectively to discuss past decisions in order to identify ways that the decision-making could have been improved. It can also be used prospectively in hypothetical future public health practice (such as “table top” exercises in emergency preparedness), in real time decision-making, and in conflict resolution of controversial public health issues, such as fluoridation of community water supplies [14].

As the process of developing these various international resources indicate, discussion of case scenarios or hypotheticals can be an effective technique in ethics education and the case analysis method of pedagogy can be an effective tool for ethics educators in public health. Cases provide a specific way to connect the scientific details and social context of public health policies and practices with more general ethical principles, rules, and values. Cases provide learners with a focus for their own ethical assessment and evaluation for decisions and actions that they might confront in their work. Cases also provide learners to reflect on past actions by professionals and to reassess them in light of various ethical perspectives.

If cases are to function educationally as described above , it is important that both experienced public health practitioners and those trained in ethical theory collaborate in the development of teaching cases and implementing courses. Cases can also serve as a resource for more general discussions of public health values among the general public. Public health ethics for professionals and for citizens in a democratic society can converge, and cases are one important bridge that can facilitate a common understanding of ethical issues.

### A strategy for ethics education in public health

Lee and colleagues report only modest progress over recent decades in incorporation of PH ethics studies in educational programs such as MPH in the United States [15]. Lee and colleagues state, “Fully integrating ethics instruction through formal coursework, infusion across curricula, and providing ethics boosters in the workplace can move us closer toward improving the ethics education of the public health workforce”. A parallel study in Europe [16], discussed by Royo Bordenado and colleagues elsewhere in this issue [17], showed similar findings.

Training current faculty on PH ethics issues should be encouraged by schools of public health in order to incorporate ethics topics into existing courses of formal educational programs. Philosophical expertise in ethics should go hand in hand with expertise and experience in professional public health practice. The teaching of ethics requires interdisciplinary competence on the part of instructors. Ethical theory and conceptual analysis are essential in the teaching of practical ethics. Moreover, integrating ethical theory into educational programs for students and practicing professionals requires careful preparation and planning. It is important to develop critical reasoning about philosophical concepts, otherwise ethical principles and norms are only used in a somewhat mechanical and rote fashion, and become an additional jargon that practitioners use rather than effective tools for analysis and decision-making.

Many ethics educators in public health find group discussions of ethics and values frustrating and difficult because such discussions seem to have no sense of progress and forward movement and no satisfying resolution or conclusion. Yet, ethics discussions need not be like this; they can—and should—follow an orderly progression of steps. And they can end, if not always with firm, agreed-upon conclusions, then at least with the sense that something has been clarified—people have been led to think.

The goals of ethics education in public health remain a subject of controversy. It should not be assumed that ethics education will automatically produce public health professionals who are more “ethical” in their personal practice than they would be 'without such instruction. Moreover, while professional ethics codes, rules, and regulations are very important, typically ethics education does not focus on them alone, and the purpose of ethics education should not simply be to familiarize practitioners with such documents. Instead of being merely formalistic or legalistic in this way, most public health ethics courses today adopt an analytic approach and focus on complex situations where numerous ethical rules and values come into play and may conflict. These involve ethical “dilemmas” or socially-politically sensitive issues — questions about which reasonable persons of good can (and do) disagree. Some of these situations involve legal and regulatory functions meant to protect individuals and societies from avoidable injury, illness or premature death.

The use of cases for discussion facilitates the development of these analytic reasoning skills because they can readily exemplify trade-offs among important values and interests and take seriously initially counter-intuitive positions. Cases involving child health and parental decision-making rights, for instance, can portray situations in which parental decisions can endanger their own or other children, such as in withholding immunizations [18], or parental refusal of vitamin K for newborns rsulting in preventable bleeding disorders, as reported in Tennessee in 2013 leading to brain damage in 4 children including one death [19].

In sum, the study of public health ethics is an essential element of preparation of practitioners and policy makers to address population health issues. This needs to address the balance between community rights and individual rights, the ethics of intervention and non-intervention with evidence-based public health practice at high standards of “best practices” as established by scientific data and epidemiological analysis of population health needs.

The aim of ethics education in public health should be to enhance the ability of public health professionals, policy makers and citizens to reason intelligently about the moral dilemmas and value conflicts inherent to human rights, social justice, and the application of knowledge and technology in the health sciences. In pursuit of this aim, ethics education in public health should attempt to stimulate the moral imagination and broaden critical reasoning skills; ethics education should elicit a recognition and sense of personal and civic ethical responsibility; and ethics education should develop a capacity to cope with moral ambiguity and to value tolerance and social diversity in a pluralistic society. Even while we must face up to disagreement and widespread misinformation, ethics education must also attempt to locate and clarify the sources of disagreement, and false science, such as allegations that the measles’ vaccine causes autism, to resolve ambiguities as far as possible, and to seek ways to overcome lack of knowledge, as well as differences of entrenched viewpoints and beliefs.

Group discussion built around the ethics cases can model this form of deliberative communication. Directed group discussion can demonstrate that progress can be made in reducing disagreement, or at least in gaining a narrower, and perhaps more manageable, area of disagreement. Such discussions can, and often do, lead to a manageable way forward. It is important to emphasize that ethics education is primarily a discipline of reasoning, not a corrective for deficient ethical motivation or character. Improved reasoning ability as a goal of ethics education does not presuppose any specific moral belief or conclusion, but it does require recourse to general standards of logic, consistency, and empirical claims that are supported by reasonable standards of evidence. Such general standards can be used to evaluate the quality of ethical arguments.

### From professional to civic education in public health ethics

When discussing a strategy for ethics education in public health it is important to recognize the audience of ordinary citizens and community members who might participate in deliberations and discussions about ethical issues alongside public health experts and practitioners. Indeed, no discussion of ethics education would be complete without taking seriously the need for civic education to complement it or to prepare social acceptance of public health initiatives that may be offensive to some but have a solid base of effectiveness, safety and cost -benefit.

To meet looming health needs, attend to public health crises, and to bring about requisite institutional and behavioral change, public health cannot rely on legal authority or coercion alone to improve population health, it must engage and persuade private individuals, who are considering their individual health interests, and public citizens, who are considering health justice for the entire community [20, 21],. Civic education concerning public health ethics builds on the findings that the fabric of social capital, individual capability, and strong social networks are integral parts of an overall pattern of living that is empowered, respected, just, and healthy.

In practice this means that public health programs must have a base in social values and purposes such that the members of these societies will understand as interdependent and relational democratic citizens [22]. As we have noted, the use of case hypotheticals in civic forums and other kinds of public discussions can facilitate better understanding. For professionals, cases can bridge the gap between facts and values. For citizens, the discussion of cases can broaden the horizons of their own understanding of their personal interests and their own place in a broader community of shared health vulnerability.

Cases shaped by background expertise in epidemiological research and current understandings of the social determinants of health can be devised which will translate statistical knowledge into culturally recognizable narrative and local knowledge. In both its professional and civic settings, public health ethics education is not about the abstractions of philosophical debate; PH ethics concerns concrete issues of health and social justice and the obligation to save lives, prevent harm, and promote the health and well-being of all people and must take into account the context of community acceptability [23].

Clearly public health involves many stakeholders and actors, including national and state governments, local authorities, advocacy groups, insurance systems, professional associations, academic centers, food and drug manufacturers, health care providers, scientists, regulators, non-governmental organizations, international agencies and donors. All are participants and contributors to what has been called “The New Public Health”. [24] While governments are crucial to leadership in public health, the practicalities and the ethics involve all of these stakeholders who all have viewpoints on the frequently controversial aspects of public health policy and practice [25].

## Conclusions/Recommendations

In this article we have described current resources available to support the teaching of public health ethics. We have argued that interdisciplinary expertise is important to the subject matter and pedagogy of public health ethics. We have discussed the role and advantages of using case studies method in ethics training. And we have suggested that ethics discussions built around cases are valuable both for public health professionals and for citizens who in a democracy are the ultimate source of authority and support for public health.

In conclusion we offer the following recommendations and action items to implement an interdisciplinary and case-based approach to ethics education.

1. Ethics should be incorporated in all courses in public health as well as health policy and management programs.
2. In addition, dedicated courses in ethics should be included in public health education curricula to provide interested students with an opportunity for more in-depth study.
3. PH ethics along with PH law should be included in criteria of accreditation agencies, such as the new European Agency for Accreditation (EPHEA) established in 2011 [26].
4. PH ethics orientation workshops should be provided to help teachers in all topic areas of the curriculum, core and elective, incorporate ethics in their teaching material.
5. The topic of PH ethics should be incorporated in ongoing educational programs for practitioners in the broad multi-disciplinary fields of public health.
6. Public awareness and engagement efforts that accompany public health programs and interventions should incorporate some measure of ethics education. Critical thinking about the values involved in a public health controversy is vital to combat the public health problem in question.
7. Recognition that the concepts of social solidarity and obligations as well as individual rights are fundamental in public health practice.
8. Emergency preparedness, voluntary or mandatory vaccination programs (e.g., should measles vaccination be mandatory for school attendance), self care, and chronic disease management programs are important current examples and there are many more.
